# Supplementary material for: Trends of Dietary Intakes and Metabolic Diseases in Japanese Adults: Assessment of National Health Promotion Policy and National Health and Nutrition Survey 1995–2019
Source: J Clin Med. 2022 Apr 22;11(9):2350. doi: 10.3390/jcm11092350 (PMC9100344; doi:10.3390/jcm11092350)
Supplement: Supplementary file 1 [file jcm-11-02350-s001.zip › jcm-1686029-supplementary.pdf]

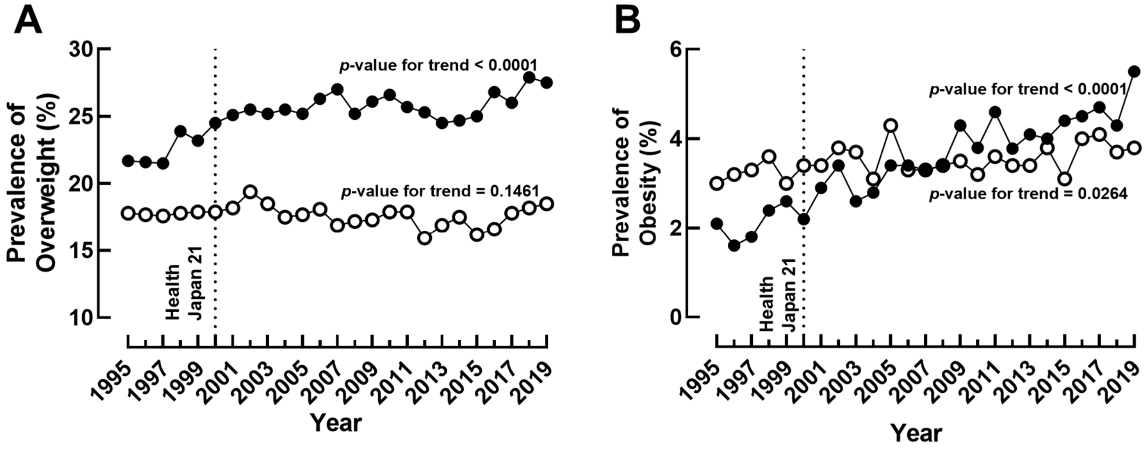

Figure S1. The prevalence of (**A**) overweight and (**B**) obesity from 1995 to 2019 among males (●) and females (○).

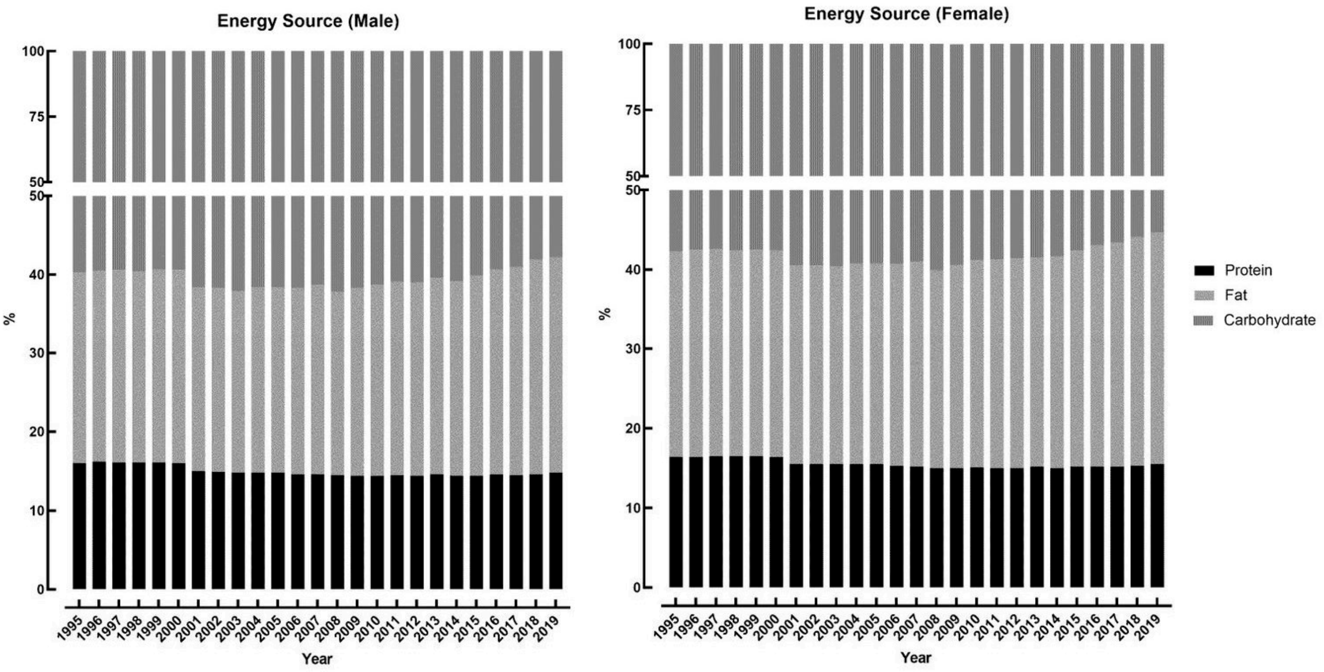

Figure S2. The proportion of energy derived from protein, fat, and carbohydrates from 1995 to 2019.

Table S1. Sample size and characteristics of the males aged ≥20 years participating in physical condition examination, blood examination, and nutrient intake assessment components of the NHNS, Japan 1995–2019.

**Sample Size**	**1995**	**1996**	**1997**	**1998**	**1999**	**2000**	**2001**	**2002**	**2003**	**2004**	**2005**	**2006**	**2007**	**2008**	**2009**	**2010**	**2011**	**2012**	**2013**	**2014**	**2015**	**2016**	**2017**	**2018**	**2019**
Total (Physical condition examination), n	4895	5051	4769	5069	4655	4513	4507	4278	3370	2749	2666	2912	2940	3027	2958	2784	2610	9781	2767	2760	2507	10594	2350	2417	1979
20-29 y.o., %	14.5	15.9	16.2	14.6	15.2	15.0	13.0	12.6	11.5	10.8	10.7	9.4	8.1	8.7	8.3	8.1	7.9	7.8	9.5	6.4	6.9	7.2	7.1	7.2	6.8
30-39 y.o., %	19.0	16.0	14.6	16.4	16.0	15.2	15.6	15.4	15.2	15.3	14.1	15.8	15.4	13.1	13.9	14.1	14.4	13.7	11.9	11.8	12.0	12.3	11.1	11.2	9.0
40-49 y.o., %	21.6	20.4	20.2	18.9	17.3	17.1	17.7	15.8	15.0	14.2	14.0	14.3	15.9	13.1	16.2	14.5	14.3	14.5	14.7	14.1	15.2	16.1	16.1	16.9	15.3
50-59 y.o., %	18.3	18.2	20.0	18.9	20.7	20.2	20.6	20.8	19.1	19.8	18.5	20.1	18.3	18.1	17.8	16.5	16.7	14.8	14.6	15.4	15.1	15.1	14.6	15.1	14.7
60-69 y.o., %	15.5	17.4	16.4	17.8	17.2	18.3	17.8	18.7	19.8	20.8	20.7	18.4	21.1	22.5	21.6	23.6	21.0	23.4	21.8	24.1	23.5	23.0	21.5	21.0	22.7
≥70 y.o., %.	11.1	12.2	12.7	13.4	13.7	14.1	15.2	16.6	19.6	19.1	22.1	21.9	21.3	24.4	22.2	23.2	25.7	25.8	27.6	28.0	27.2	26.3	29.7	28.5	31.4
BMI Measurement, %	85.3	82.6	84.7	84.7	75.9	83.4	80.2	79.1	99.7	99.7	99.2	99.8	99.6	99.5	98.8	98.1	99.0	99.0	99.0	99.5	98.8	79.9	99.2	99.3	98.8
BP Measurement, %	60.2	57.2	59.2	60.3	51.1	56.0	50.6	52.5	66.1	59.8	61.6	63.6	59.0	63.5	61.0	61.0	60.7	63.0	53.7	56.3	57.2	46.9	55.0	53.2	55.0
Total (Blood examination), n	1710	2208	2506	2622	2055	2277	2132	2129	2112	1549	1558	1753	1625	1819	1737	1598	1463	5775	1382	1476	1337	4659	1213	1204	1020
20-29 y.o., %	16.9	N/A	9.9	10.6	8.5	9.2	7.4	6.9	7.7	7.6	7.6	6.6	5.5	5.6	5.2	4.7	5.0	5.2	6.6	3.5	4.3	4.2	4.8	4.3	5.4
30-39 y.o., %	25.5	15.3	13.1	14.5	13.3	12.3	11.4	11.9	12.5	11.4	10.1	12.1	12.3	9.8	11.4	10.1	12.4	10.9	7.9	7.3	8.5	8.6	7.4	8.6	6.3
40-49 y.o., %	28.2	22.3	18.4	16.9	15.4	14.8	17.2	13.9	12.8	11.0	11.3	11.9	12.9	10.2	13.8	11.0	10.5	11.9	10.2	10.9	11.4	12.7	10.1	12.1	11.4
50-59 y.o., %	29.4	19.5	20.1	18.6	19.1	20.9	19.0	19.9	19.0	18.3	16.6	20.0	16.3	16.1	17.1	16.2	14.7	14.3	13.0	14.8	12.3	12.8	13.1	13.4	12.6
60-69 y.o., %	N/A	25.3	22.0	22.6	24.6	24.6	24.2	25.5	24.3	26.2	25.5	22.2	25.4	26.7	25.6	28.9	24.3	26.9	26.4	28.5	28.6	27.9	26.5	25.1	24.4
≥70 y.o., %.	N/A	17.6	16.6	16.9	19.0	18.3	20.8	22.0	23.8	25.5	28.9	27.2	27.6	31.8	26.9	29.1	33.1	30.8	35.9	35.0	34.9	33.8	38.1	36.5	39.9
HbA1c Measurement, %	N/A	N/A	100	N/A	N/A	N/A	N/A	99.5	99.4	99.7	99.7	99.5	99.6	99.7	91.8	91.1	99.8	99.6	99.9	99.5	99.1	99.8	99.3	99.8	99.6
Total (Nutrient Intake Assessment), *n*	4895	5051	4769	5069	4655	4414	4438	4190	4172	3214	3351	3506	3328	3508	3359	3334	3085	12265	2998	3112	2840	9987	2714	2663	2297
20-29 y.o., %	14.5	15.9	16.2	14.6	15.2	14.9	13.0	12.3	12.4	11.0	11.9	10.5	9.1	9.6	8.8	9.3	8.4	8.8	9.1	7.0	7.9	7.1	8.1	7.9	8.0
30-39 y.o., %	19.0	16.0	14.6	16.4	16.0	15.1	15.4	15.4	16.1	16.3	15.2	16.6	16.2	13.6	14.9	15.2	15.5	14.0	12.3	12.1	12.2	12.1	11.9	11.8	9.1
40-49 y.o., %	21.6	20.4	20.2	18.9	17.3	17.1	17.7	15.8	15.4	14.9	14.9	14.8	16.1	14.2	16.0	15.5	15.6	15.0	15.2	14.8	16.0	15.8	17.0	16.7	15.3
50-59 y.o., %	18.3	18.2	20.0	18.9	20.7	20.3	20.8	20.9	19.6	20.2	18.6	20.0	17.6	17.5	18.0	16.2	16.9	15.2	15.2	15.3	15.5	14.9	14.4	15.7	15.2
60-69 y.o., %	15.5	17.4	16.4	17.8	17.2	18.4	17.9	18.9	18.5	19.6	19.3	17.6	20.0	21.5	20.5	21.8	19.4	22.5	21.3	23.8	22.5	23.1	20.9	20.6	21.9
≥70 y.o., %.	11.1	12.2	12.7	13.4	13.7	14.1	15.1	16.7	18.1	17.9	20.1	20.5	20.9	23.6	21.7	22.0	24.2	24.6	27.0	27.0	25.9	27.0	27.8	27.3	30.5
Occupation																									
Professional/Manager, %	62.8	60.0	60.5	59.1	56.3	58.5	21.4	21.4	20.2	20.9	22.4	20.1	22.1	20.1	20.3	22.0	20.3	N/A	20.3	18.8	15.3	20.9	21.5	21.6	21.1
Sales/Service/Clerical, %	13.2	13.0	14.0	13.2	14.0	11.8	22.3	22.9	21.2	22.7	21.8	21.4	21.1	20.2	20.8	21.1	20.8	N/A	22.1	20.4	15.8	20.2	20.0	23.5	19.0
Security/Transportation/Labor/Agriculture/Forestry, %	5.8	7.6	6.0	6.0	7.0	6.5	36.5	35.1	36.2	32.8	32.5	35.1	32.2	32.8	29.9	30.3	29.9	N/A	25.5	29.5	38.7	33.2	28.5	27.1	30.5
Housewife/husband, %	0.1	0.2	0.1	0.1	0.1	0.2	1.3	1.5	1.1	1.0	1.5	1.1	1.2	1.5	2.9	1.8	2.9	N/A	2.5	2.1	2.6	1.3	3.2	2.6	2.9
Non-worker, %	18.0	19.3	19.4	21.5	22.6	23.1	18.4	19.2	21.2	22.7	21.9	22.3	23.4	25.4	26.1	24.8	26.1	N/A	29.6	29.1	27.7	24.5	26.8	25.2	26.5

Table S2. Sample size and characteristics of the females aged ≥20 years participating in physical condition examination, blood examination, and nutrient intake assessment components of the NHNS, Japan 1995–2019.

**Sample size**	**1995**	**1996**	**1997**	**1998**	**1999**	**2000**	**2001**	**2002**	**2003**	**2004**	**2005**	**2006**	**2007**	**2008**	**2009**	**2010**	**2011**	**2012**	**2013**	**2014**	**2015**	**2016**	**2017**	**2018**	**2019**
Total (Physical condition examination), n	5791	5814	5637	5852	5465	5163	5318	4995	4262	3439	3316	3622	3601	3743	3670	3442	3191	12265	3263	3256	3082	12496	2756	2817	2355
20-29 y.o., %	16.3	16.3	16.4	14.0	15.5	13.4	13.1	12.2	10.6	10.6	9.1	9.2	8.4	7.9	8.1	7.7	8.4	7.1	7.7	7.3	6.9	6.8	6.0	6.6	5.9
30-39 y.o., %	17.0	15.0	14.1	15.9	15.0	15.2	15.9	14.5	15.0	15.4	13.7	16.3	16.7	13.7	14.2	14.2	14.1	13.3	12.4	11.3	10.8	11.5	10.3	11.1	9.6
40-49 y.o., %	20.0	19.2	18.9	17.8	16.3	16.9	16.3	14.9	14.7	14.7	14.8	13.8	14.8	12.9	15.0	14.3	14.2	14.3	15.5	14.7	17.4	15.6	16.0	15.1	15.2
50-59 y.o., %	17.9	17.8	18.9	19.0	18.9	20.3	19.4	19.9	19.5	19.6	18.8	19.7	17.9	18.5	16.6	16.6	15.3	15.8	14.2	15.8	15.5	15.0	14.8	16.2	16.1
60-69 y.o., %	14.8	17.2	15.3	16.8	17.5	16.5	17.1	17.7	19.3	20.2	20.9	18.6	20.1	21.4	21.2	22.4	21.1	22.7	22.5	23.1	22.9	22.3	21.0	21.3	21.4
≥70 y.o., %.	14.1	14.4	16.4	16.6	16.8	17.5	18.2	20.8	20.9	19.5	22.7	22.4	21.9	25.8	25.0	24.7	26.9	26.7	27.7	27.8	26.5	28.7	31.9	29.7	31.8
BMI Measurement, %	89.0	87.6	89.5	89.0	82.7	87.3	86.2	84.2	98.9	99.0	98.7	98.9	98.8	98.9	98.7	97.7	98.1	98.4	98.0	98.8	98.6	83.0	98.3	98.4	98.4
BP Measurement, %	76.6	73.3	74.6	76.3	66.8	70.0	67.7	67.0	79.3	73.1	73.3	75.0	71.1	73.3	72.4	70.4	70.9	72.9	63.6	66.0	68.4	57.2	66.2	65.5	64.2
Total (Blood examination), n	2827	3422	3803	3943	3205	3288	3385	3183	3195	2383	2316	2565	2393	2632	2556	2275	2094	8374	1919	2028	1983	6732	1711	1744	1411
20-29 y.o., %	17.9	N/A	11.6	10.4	11.2	8.5	8.6	7.8	7.6	8.0	6.7	6.6	4.9	5.5	5.5	4.6	6.1	5.2	5.5	4.2	4.3	4.0	3.9	4.8	3.3
30-39 y.o., %	24.8	17.2	15.0	16.6	15.3	15.2	15.4	14.2	14.5	14.7	12.6	15.4	16.3	12.8	13.2	14.2	13.0	12.3	11.4	10.7	10.5	10.3	9.2	10.8	8.2
40-49 y.o., %	29.4	23.1	19.0	17.2	16.0	2.2	17.5	14.8	14.9	14.6	14.2	14.2	14.5	12.0	14.7	13.5	13.2	13.7	14.8	13.7	16.6	14.0	15.1	13.2	15.0
50-59 y.o., %	27.9	21.4	20.8	20.8	19.6	22.6	20.2	21.7	20.8	20.5	19.6	20.5	18.8	19.1	17.6	17.2	15.7	16.5	14.2	16.8	15.5	14.8	15.0	15.8	15.7
60-69 y.o., %	N/A	22.7	18.1	19.1	21.0	19.2	20.4	20.9	21.6	22.4	23.2	20.5	21.9	23.7	22.8	24.9	23.6	24.9	25.5	25.0	25.8	26.2	22.9	23.0	23.4
≥70 y.o., %.	N/A	15.5	15.4	16.0	16.8	17.1	17.9	20.6	20.6	19.7	23.7	22.7	23.6	26.9	26.1	25.5	28.4	27.4	28.6	29.5	27.3	30.7	33.8	32.5	34.6
HbA1c Measurement, %	N/A	N/A	100	N/A	N/A	N/A	N/A	99.3	99.7	99.6	99.7	99.5	99.6	99.6	95.2	94.8	98.7	99.6	99.5	99.6	99.6	99.8	99.5	99.4	99.6
Total (Nutrient Intake Assessment), *n*	5791	5814	5637	5852	5465	5095	5269	4930	4792	3823	3911	4085	3881	4136	4018	3895	3668	14461	3483	3615	3332	11864	3080	3080	2630
20-29 y.o., %	16.3	16.3	16.4	14.0	15.5	13.3	13.0	12.1	11.5	11.8	10.3	10.1	9.3	8.8	9.0	8.7	8.9	8.1	8.2	7.5	7.4	6.6	6.5	7.0	6.9
30-39 y.o., %	17.0	15.0	14.1	15.9	15.0	15.3	15.9	14.4	15.1	15.7	13.9	16.4	17.0	13.7	14.4	14.8	14.3	13.4	12.1	11.6	10.9	11.4	10.3	11.5	9.5
40-49 y.o., %	20.0	19.2	18.9	17.8	16.3	17.0	16.4	15.0	14.4	14.8	15.1	14.2	14.7	13.0	14.8	14.4	15.2	14.6	15.3	15.2	17.5	15.3	16.8	15.3	14.9
50-59 y.o., %	17.9	17.8	18.9	19.0	18.9	20.5	19.5	20.2	19.1	19.0	18.5	19.1	17.5	18.2	16.2	16.1	15.1	15.6	14.5	15.2	15.6	15.0	15.3	15.9	16.2
60-69 y.o., %	14.8	17.2	15.3	16.8	17.5	16.5	17.2	17.8	18.2	19.3	19.9	17.7	19.6	20.8	20.3	21.4	20.4	21.4	22.1	22.3	22.0	22.3	20.1	20.3	20.7
≥70 y.o., %.	14.1	14.4	16.4	16.6	16.8	17.4	17.9	20.6	21.8	19.5	22.3	22.4	21.8	25.4	25.3	24.7	26.0	26.9	27.8	28.1	26.7	29.5	31.0	29.9	31.9
Occupation																									
Professional/Manager, %	30.6	29.5	30.1	30.9	28.8	29.3	22.0	19.0	21.0	22.2	22.7	21.0	22.5	20.1	21.5	21.1	21.5	N/A	21.5	20.1	6.6	20.6	24.3	25.1	25.0
Sales/Service/Clerical, %	6.3	5.8	7.3	7.1	6.0	5.8	15.5	16.5	16.1	17.1	18.0	17.0	17.4	15.1	18.9	17.8	18.9	N/A	17.9	18.3	23.2	17.8	17.0	17.6	17.1
Security/Transportation/Labor/Agriculture/Forestry, %	6.6	7.6	6.9	4.1	7.8	7.6	11.7	12.3	11.7	9.1	9.0	10.9	8.9	10.0	7.4	7.3	7.4	N/A	6.7	8.2	17.7	9.3	7.4	7.5	9.6
Housewife/husband, %	40.4	41.7	39.3	40.9	40.3	39.7	40.7	41.4	39.4	39.7	39.4	38.3	40.5	42.4	41.3	41.3	41.3	N/A	41.5	40.8	40.5	39.7	39.1	37.2	36.8
Non-worker, %	16.1	15.4	16.4	17.0	17.2	17.5	10.1	10.8	11.8	11.9	10.9	12.7	10.7	12.4	10.9	12.5	10.9	N/A	12.3	12.5	12.1	12.6	12.2	12.7	11.5
